# Montelukast for the high impact of asthma exacerbations in Venezuela: a practical and valid approach for Latin America?

**DOI:** 10.1186/1939-4551-7-20

**Published:** 2014-09-04

**Authors:** Arnaldo Capriles Hulett, Maria Gonzalez Yibirin, Amaris Garcia, Dollys Hurtado

**Affiliations:** 1 Centro Médico de Caracas; Centro Médico Docente La Trinidad y Programa de Medicina Comunitaria; Allergology Unit, Hospital San, Juan de Dios, Caracas, Venezuela; 2 LETI Laboratories, Caracas, Venezuela; 3 General Practitioner, ambulatory health care facility “Los Erasos”; Health District 1, Ministry of Health, Caracas, Venezuela; 4 Programa de Medicina Comunitaria, Centro Médico Docente, La Trinidad, Venezuela

**Keywords:** Asthma, Asthma exacerbations, Asthma control, Montelukast, Practical approach, Deprived urban majorities, Venezuela, Latin America

## Abstract

**Background:**

Asthma affects mainly Venezuela’s urban and poor majority. Exacerbations bring about a high demand in health services, thus becoming a significant public health problem. In general, asthma control programs (GINA) with use of inhaled steroid medications have proven effective, although their implementation in real life remains cumbersome. Montelukast could be a useful and practical tool for these deprived socioeconomic sectors.

**Methods:**

This real-life pilot study was conducted in a prospective, double blinded, placebo-controlled manner with randomized and parallel groups. Asthmatics that had never used leukotriene modifiers were recruited and followed-up every three months. The main outcome was the number of exacerbations meriting use of nebulized bronchodilators administered by the health care system.

**Results:**

Eighty-eight asthmatic patients were enrolled, between children and adults. Groups were comparable in: demographic data, previous use of other medications, ACT scores, pulmonary functions (*Wright Peak Flow meter*), allergy status (*Skin Prick Test*) as well as adherence to the prescribed Montelukast treatment. By an intention to treat (ITT), a total of 64 patients were included for analysis. For the three and six months time points the difference between placebo and Montelukast was found to be significant (p < 0.03 and p < 0.04, respectively). Such trends continued for the rest of the year, but without statistical significance, due to patient attrition.

**Conclusions:**

This real-life pilot study shows that a simplified strategy with oral Montelukast was practical and effective in controlling exacerbations in an asthmatic population of a vulnerable community from Caracas. Such an approach reinforces the role of primary care in asthma treatment.

## Introduction

Asthma in Venezuela is a public health problem, derived from its high prevalence and significant impact due to recurrent exacerbations [1-5]. About one third of asthmatics share severe asthma characteristics [6]. Venezuela’s population is predominantly urban (90%) and approximately half of it [7] lives under variable conditions of poverty (Graffar Scale’s^a^ D and E). Asthma prevails in such deprived socioeconomic sectors [8] and a recent study of a vulnerable population in Caracas, reflecting its urban and poor majority, supports such findings [9].

The increase in exacerbation rates for the past 20 to 25 years, represent asthma’s major impact; one million acute episodes/year for 28 million inhabitants [2] is compelling evidence (data from the Ministry of Health - MoH - ambulatory network, caring for 80% or more of the population). Furthermore, in such settings, asthma exacerbations are often found second only to the febrile syndrome and rarely overtaken by consultations for diarrheas [2].

An existing National Asthma Control Program [1,2] patterned after the Global Initiative for Asthma (GINA) guidelines was last revised in 1998, but weakly implemented over the years. Inconveniences related to the use of inhaled medications [10] make, among other considerations, a simplified asthma approach worth pursuing [11]. In analogy to oral rehydration therapies in third world countries [12], use of an oral and for the most part innocuous medication (Montelukast, MLK ), seems particularly attractive for Venezuela’s urban and poor majority context [8,9,11].

To address the impact from exacerbations a real-life pilot project with Montelukast was carried-out, in a double blinded placebo-controlled manner for six months, to be further prolonged over a year-long period. Adherence [10], educational needs, tobacco smoke exposure [13] and sustainability in time were among the many issues considered. Inaction is not an option regarding the social determinants of asthma [10,14].

## Materials and methods

**Figure 1:**
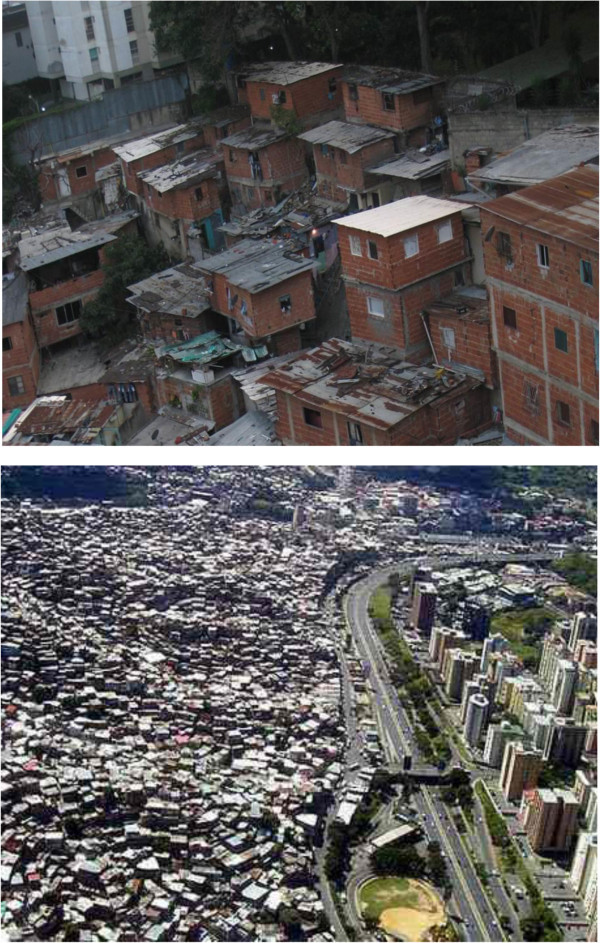
**The first photo, taken from the nearby private medical facility, Centro Médico de Caracas, portrays living conditions of the studied community.** The second, an aerial photo, (below) provides a better insight into these aspects of Caracas.

This real-life pilot study was registered and audited under number 090267 by the clinical trials department of the *Instituto Nacional de Higiene Rafael Rangel,* an official organization of the Venezuelan MoH. The Institutional Review Board of “*Centro Médico de Caracas*” also approved this study. Funding was obtained from LETI laboratories, in Caracas, Venezuela. The study was, double blinded, randomized, and run in parallel groups. Patients, including children with their representatives, signed a consent form regarding the scope of the study and the use of placebos. The “Los Erasos” slum was chosen (see Figure 1) because it possessed [9] the living conditions of 50% of the inhabitants of Caracas, the capital city of Venezuela, and is located near the private medical center. In addition, this community demonstrated in a previous evaluative survey, the relationship between asthma and poverty. A high prevalence of physician diagnosed asthma (15%) was found among its inhabitants, with 2/3 of these patients in a non - control status as determined by the ACT, for both children and adults [9].

Asthma diagnosis also carried a recent history of frequent extra-domiciliary nebulizations for acute symptoms relief. The existing health care system [2] provides routine rescue treatments dispensed with nebulizations of a Fenoterol/Ipatropium bromide inhalation solution (0.25 mgs/0.5 mgs) at an age adjusted dosing (10 gtts children - 20 gtts adults), diluted in 2–3 cc of 0.9% saline. No patients were admitted at any stage of pregnancy, nursing or having had previous use of leukotriene modifiers; however, any other therapy was allowed and recorded. If during the course of the study a patient was to become pregnant, she would have to be withdrawn immediately. Patients and their families attended voluntarily and were invited to participate in the study by means of information from slum community leaders and word of mouth from neighbors. Eagerly concerned families made for children numbers come close to half of the participants, along with very few adult asthmatic smokers.

The main variable was the number of exacerbations (primary endpoint) needing rescue nebulizations with a Fenoterol/Ipatropium combination administered at the health care system; patients in need of assistance after evening hours had to attend local hospitals nearby but outside the community. Close monitoring of the primary outcome was carried by monthly phone calls and detailed questioning during regular visits. This project was originally conceived for execution at the ambulatory health facility (MoH) located within the community; however, due to security concerns for the researchers, it was carried out in a private medical office (ACH) across the street, imposing limits on logistics particularly regarding number of participants.

A total of 88 known asthmatic patients (roughly 1/2 were children) were recruited. The patients were between 5 and 44 years of age, and were either smokers or non-smokers. Those patients who had not smoked cigarettes in the last 6 months were considered as non-smokers. The daily consumption rate and the years of smoking were recorded for those who reported tobacco use*.* Asthmatics between the ages of 5 and 12 were referred to as children, while those 13 and up to 44 years of age were defined as adults, in conformity with the ACT age ranges. These age considerations helped avoiding confusion with other respiratory syndromes, such as early ages viral wheezing and chronic obstructive pulmonary disease in the elderly. This does not imply however, the existence of asthma outside of these age ranges.

Asthmatics underwent a detailed clinical history and physical examination (with an emphasis on co-morbidities such as allergic rhinitis and flexural atopic dermatitis). Weight, height, body mass index, pulse and blood pressure, together with prick skin tests (Lancetters, Hollister-Stier) for inhalant allergens (Greer Labs, ALK-Abello) were recorded during the examination. The best of three standing Wright Peak Flow Meter measurements in L/min (Ferraris Medical, Holland, New York, USA), not varying by more than 5% and performed before and 20 minutes after 400 mcg of albuterol, were obtained. Patients were administered the ACT test according to age and received minimal oral information and a simple written one-page educational pictorial document.

Children received a 5 mg dosage of Montelukast daily while adults received a 10 mg daily dosage. Montelukast and placebo treatments were supplied in aluminum blister packs (chewable tablets for those 4–15 years of age, 10 mgs tablets for those 15–44 years of age), to be taken at night prior to going to bed. The counting of empty blister packs allowed for the estimation of adherence on return visits.

Visits were scheduled every 3 months and patients received a monthly phone call. During those calls they were reminded to take the prescribed medication and to follow recommendations on environmental control. Likewise, emphasis was placed on the date of the next hearing and on returning the blister aluminum packs for proper evaluation of adherence. Furthermore, and most importantly, they were urged to take note of exacerbations that merited medical care (extra domiciliary) from the local health care system.

This information was requested on every visit and collated with the data obtained from the monthly phone calls. Patients were allowed to take any other suitable medications to treat their asthma (except leukotriene modifiers), in an attempt to reflect a real life situation; no further recommendations were given. During follow-up visits, patients were questioned as to whether their rhinitis had improved, had remained the same or had worsened, as an indirect means of evaluating adherence.

### Statistics

The sample size was calculated using as a reference a study [15] of Infliximab vs placebo (Erin EM, Leaker BR, Nicholson GC et al. **The effects of a monoclonal antibody directed against tumor necrosis factor-alpha in asthma**. *American Journal Respiratory Critical Care Medicine 2006*; **174** (7): 753–762). This study compared the number of exacerbations (primary endpoint) in asthmatic patients resulting in 72% of exacerbations for the placebo group vs 29% for the Infliximab group. We needed at least 30 patients per group to show a difference between the placebo and the Montelukast treated group for a power of 80%. The percentage of patients with acute exacerbations (primary end point) was evaluated by the Chi square test. Alpha error level for all variables was 5%.

## Results

From the onset, the groups were comparable with regards to the use of asthma medication, ACT test scores, WPFM measurements and demographic data (age, sex, weight, height, BMI, blood pressure and pulse). No patient had previously used MLK; forty-six asthmatics comprised the medication and forty-two the placebo group (Table 1).

**Table 1:** Demographics

	**Montelukast**	**Placebo**	**p**
Patients N°	46	42	
Age	18,27+/-14,14	16,88+/-13,81	0,69*
Sex F/M	18/17	27/15	0,93**
Weight	47,16+/-25,11	45,96+/-28,49	O,91*
BMI	21,48+/-6,45	21.30+/_8,13	0.99*
Mite allergy	56.52 %	47.62 %	0.74**
Allergic Rhinitis	78.26 %	63.08 %	0.71**

*t student.

**Chi^2^.

During the intended first six months, the number of patients allowed for significant statistical analysis, if children and adults were grouped together. The loss of patients, whereby only 19 of the initial 88 recruited patients completed the extended year-long treatment, did not permit this.

Approximately 57% of the asthmatic patients had a positive (> than 3 mm papule over negative control and read at 10 min) prick skin test to a mixture of mites (*Dermatophagoides pteronyssinus/Dermatophagoides farinae) as* well as to *Blomia tropicalis*, which was in agreement with what had previously been reported in Venezuela. As well, lower rates of sensitization to other inhalant allergens did not reveal (epithelia, molds, grasses, cockroach, rodents) any significant difference between the groups.

**Figure 2:**
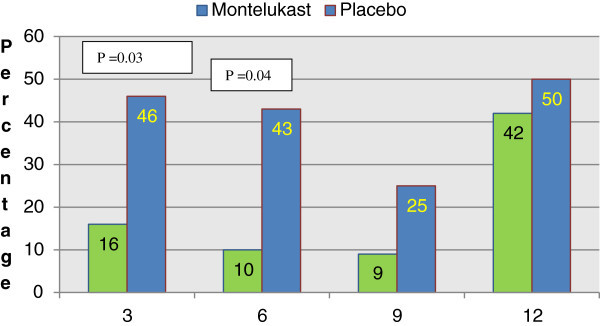
Percentage of all patients with acute exacerbations in need of rescue nebulizations at the local health system attended by the group studied.

In regards to the co-morbidities (average rhinitis diagnosis of 70% and flexural atopic dermatitis 8%) in patients and the functional aspects for the diagnosis of asthma (reversibility), no difference was observed among the groups. In the case of children however, a better response to bronchodilatadors (>20% reversibility) was shown (80%). There was a trend for improvement in the MLK group for the WPFM and ACT scores, though no significant differences between the groups were detected during the year-long study.Following analysis allowed detecting, during the first 6 months, a significant difference between the groups in the number of exacerbations needing assistance from the health care system (Figure 2). Although there was not a statistically significant difference in the second half of the study, a continued tendency for fewer exacerbations with the use of MLK, was observed. We believe this was due to the small number of patients.

Adherence to the medication was between 80% and 90% for the three and six months time points, respectively, with no significant differences observed between the groups. By adherence we meant a return of 70% or more of consumed tablets from the blister aluminum pack. Patients who received the active drug reported a significant “improvement” in their rhinitis symptoms (yes/no) in comparison with the placebo group. Three adverse effects from the use of this medication were reported. Two occurred in adults, and were mild and unrelated to the treatment (one patient had a slight foot pain and the other had an urticarial reaction that was medicated with Loratadine). One child had diarrhea, but did not require suspension of the treatment. An episode of diarrhea in an adult showed up in the placebo group, which required suspension of the treatment.

## Discussion

Asthma in Venezuela is an important public health problem [1,2,5] as evidenced by its high prevalence and significant impact due to exacerbations. We attempted to show that for Venezuelan’s urban and poor majority context a reduction in the number of exacerbations by regular use of Montelukast is an attainable goal. Venezuela’s public health care system, however, persists on the view that asthma is mainly an acute problem [9].

Venezuela has approximately 28 million inhabitants with 90% living [7] in low socio-economic urban areas which, according to a WHO operational classification, can be considered as “slums” [16]. In addition, close to 50% of the population is classified under Graffar’s D and E scale [7,17] where the greatest prevalence of asthma is found [8,9]. Furthermore, asthma is a social disease [18] demanding approaches that should not discard the varied cultural elements surrounding it.

The number of asthma exacerbations has increased significantly over the past 20 years [2,9] coming close to a million acute asthma episodes/year. This surpasses the number of exacerbations/year [19] in the USA (a population ten times higher) by more than five times. These asthma exacerbations occur primarily during evening hours when these MoH outpatient health facilities [2,9] are closed and increased security issues become more apparent. The significant absenteeism from school and work, whereby 90% of children suffering from acute asthma do not attend school and 50% of adults do not attend work, rounds up asthma’s impact [9].

Venezuela’s National Asthma Control Program based on the GINA guidelines (inhaled Beclomethasone) has been only weakly implemented and not revised since 1998 [1,2]. Moreover, data from a non-profit organization (PROVEA, 2006) points clearly for asthma to be underfunded [8,20]. Since 2004, there has been a parallel health care system run by Cubans [21], which addresses a portion of the urban and poor majorities; however, no financial or clinical information is available.

Successful asthma control strategies under the GINA guidelines have been carried out in some areas of Latin America [22]. Inconveniences around the use of steroid inhaled medications [10] make for mitigating asthma exacerbations with simpler yet effective strategies [23,24]. For these vulnerable urban and poor majorities, and in analogy to oral rehydration therapies [12] for diarrheas and dehydration, a paradigm for asthma is found wanting; this study is conceived within these limits. Incidentally, simplifying asthma treatments has been a cornerstone of some of our previous efforts [25,26].

An important aspect of this real-life pilot study was the double-blinded, placebo-controlled design with monthly telephone calls for main outcome monitoring (asthma exacerbations meriting the use of nebulized Fenoterol/Ipatropium Bromide bronchodilators in the health care system); nearly all urban households in Venezuela have a cell phone-line [27]. Similar techniques have proven useful in clinical settings [28] by increasing the degree of reliability of predetermined outcomes. Patient groups at entry were comparable in their demographics, ACT scores, atopy (positive skin prick test to mites) and co-morbidities.

An unexpected drawback however, was the high attrition rate of patients as the study progressed through the final six months. It was acknowledged from the beginning that this slum population was a hard one to study, not discarding cultural elements for the high dropout rate. The same was not carried out inside the community boundaries; to attend the doctor’s office, patients had to climb a series of long staircases, an element which may have contributed to the high attrition rate. One of the lessons learned, and an issue that must be seriously considered for future similar projects in urban and poor settings, is the need to maintain a high motivation level among local community leaders, patients and families alike. Most importantly, any such program must be conducted physically within the host community and dealt “in situ” with any issues that may arise. These aspects were not given the weight they deserved given the concerns pertaining to the researchers’ security.

The opportunity offered during an exacerbation to verbally educate and succinctly reinforce concepts with pictorially written material, should be taken advantage of. This may be the only contact an asthmatic in Venezuela has with the health care system**.** A simple rule of two’s (2′s) might serve [29] to discern which patients are in need of asthma control treatments. Focus on primary care remains pivotal when a simple approach as this is used. Adherence issues, as with any asthma interventions around the world, need to be dealt with local and cultural factors. Sustainability in relation to cost issues is an important consideration; the recent expiration of the MLK patent [30] comes in rather conveniently if bulk quantities are to be needed for large scale programs. An IMS Health report for Venezuela [31] reveals a particular and interesting reality: a combination of a brand name Fluticasone/Salmeterol 500/50 Diskus for a one month treatment is 3 times more expensive than a whole month of Montelukast 10 mgs tablets, such as the one employed in this study (US$ 16 vs. US$ 5 respectively). As a comparison, one pack of 20 cigarettes of a common local brand costs US$ 1.3 and the minimum monthly wage is set at US$ 82.5 (April 2014).

To conclude, we believe that simplifying the treatment of asthma by aiming to reduce exacerbations can be achieved with the administration of the oral medication Montelukast. If these results are reproduced, they may give a boost and allow for significant changes in the National Asthma Control Programs and thus lessen the impact that asthma exacerbations impose on the quality of life of patients and families. Given the contributory role that rhinitis [32] plays in the lack of control of asthma symptoms, the recent availability in the Venezuelan pharmaceutical market of a Desloratadine - Montelukast combination [33] in one tablet (Desler M®), prompts future research in this area. Though acknowledging the need for further study, we think these efforts point into the right direction.

## Endnote

^a^Graffar Scale is a widely applied index that divides population in five descending layers (A through E) according to a score of socioeconomic features [17].

## Competing interests

The authors declare that they have no competing interests.

## Authors’ contributions

ACH: participated in study design and patient care. MGY: participated in logistics support, study design and statistical analysis. AG: participated in patient care. DH: participated in study design and performed needed social work with the community. All authors read and approved the final manuscript.
